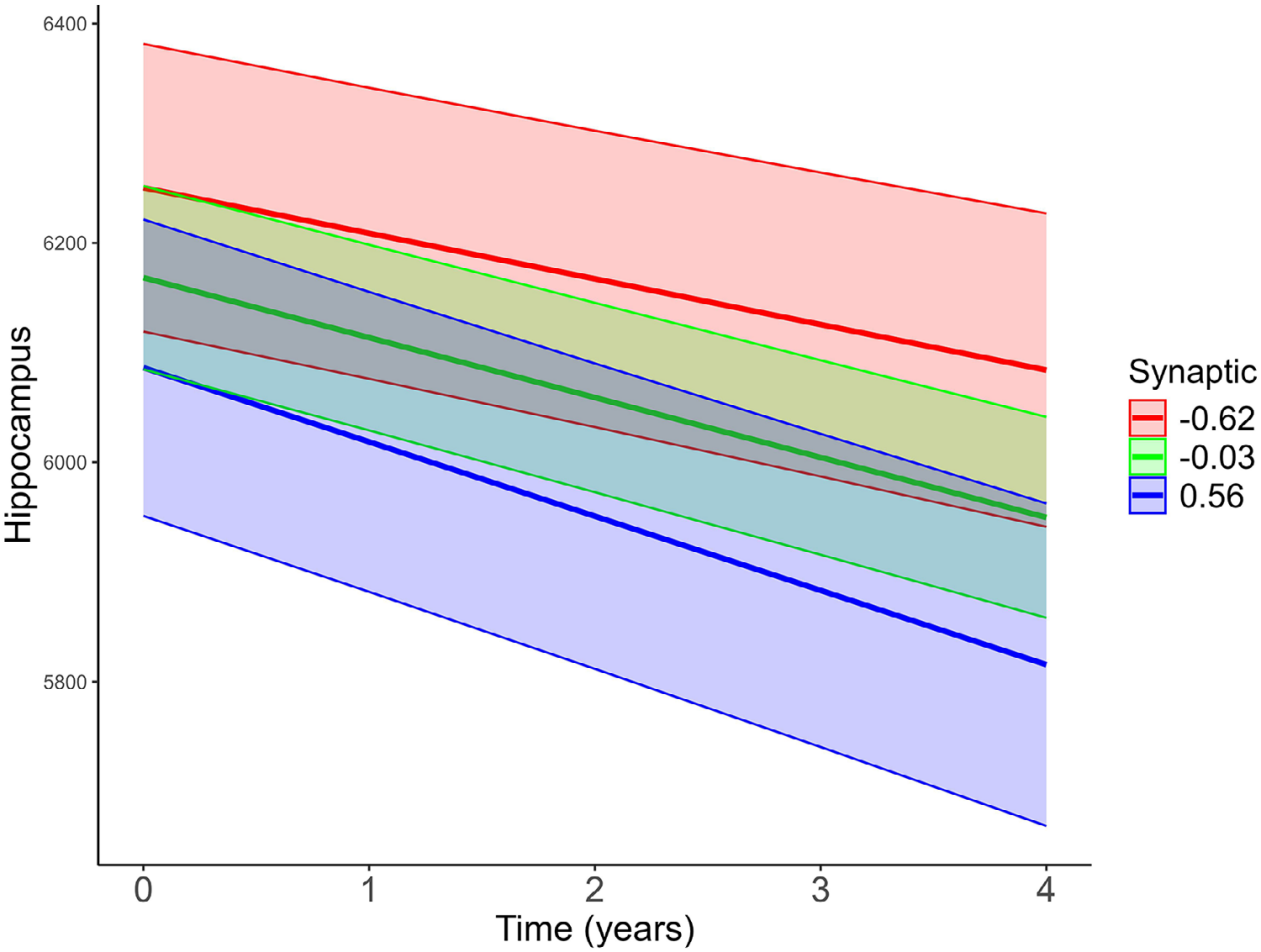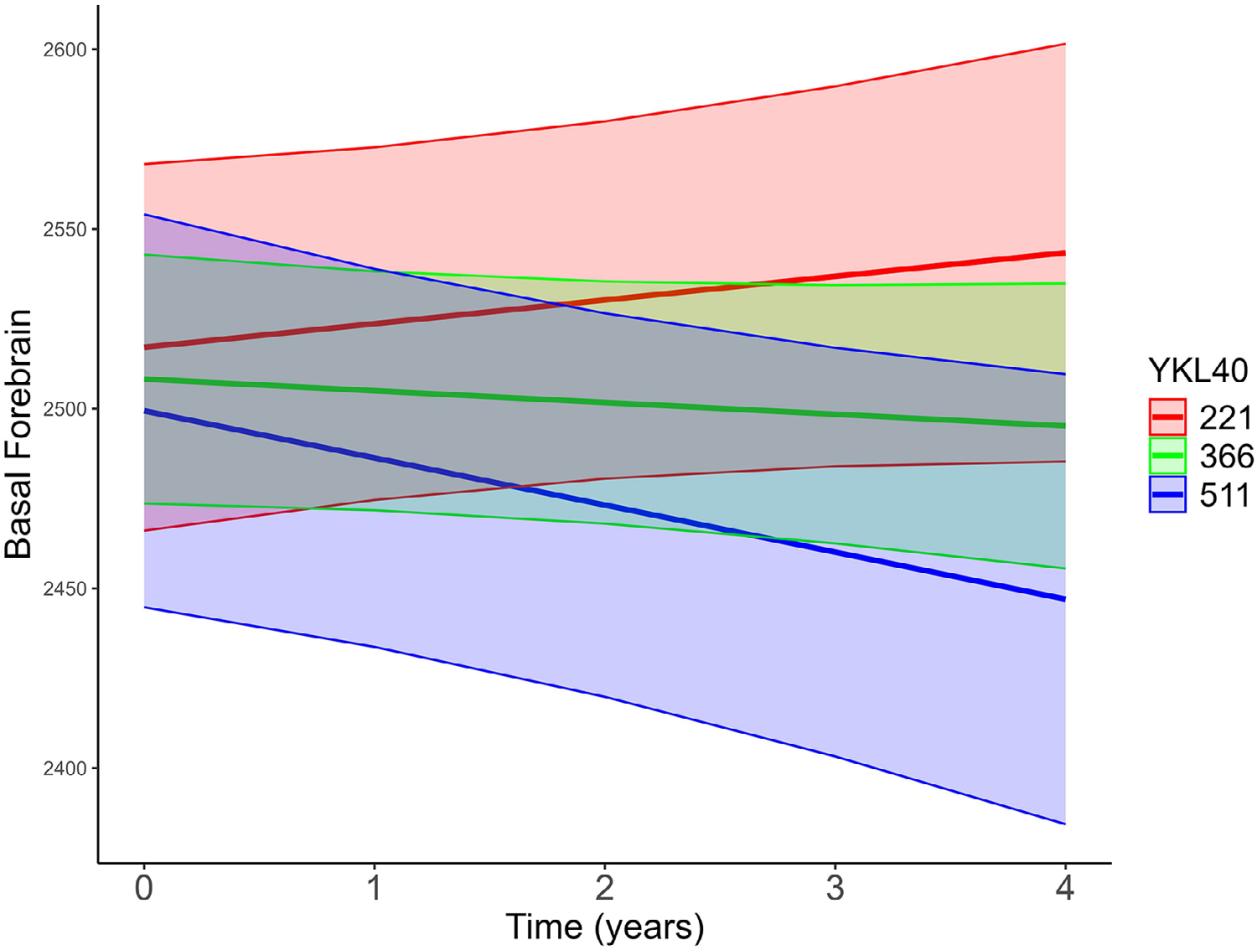# CSF Biomarkers of Neuroinflammation in Prediction of Brain Atrophy

**DOI:** 10.1002/alz70861_108159

**Published:** 2025-12-23

**Authors:** Serap Özlü, Alice Grazia, Martin Dyrba, Frederic Brosseron, Katharina Buerger, Emrah Düzel, Julian Hellmann‐Regen, Frank Jessen, Luca Kleineidam, Christoph Laske, Robert Perneczky, Oliver Peters, Josef Priller, Alfredo Ramirez, Anja Schneider, Annika Spottke, Matthis Synofzik, Jens Wiltfang, Stefan Teipel

**Affiliations:** ^1^ Deutsches Zentrum für Neurodegenerative Erkrankungen e.V. (DZNE), Rostock, Mecklenburg‐Vorpommern Germany; ^2^ Department of Psychosomatic Medicine, University Medicine Rostock, Rostock, Germany, Rostock Germany; ^3^ German Center for Neurodegenerative Diseases (DZNE), Rostock Germany; ^4^ German Center for Neurodegenerative Diseases (DZNE), Venusberg‐Campus 1, 53127, Bonn Germany; ^5^ German Center for Neurodegenerative Diseases (DZNE), Munich Germany; ^6^ German Center for Neurodegenerative Diseases (DZNE), Magdeburg Germany; ^7^ Charité – Universitätsmedizin Berlin, corporate member of Freie Universität Berlin and Humboldt‐Universität zu Berlin – Institute of Psychiatry and Psychotherapy, Berlin Germany; ^8^ German Center for Neurodegenerative Diseases (DZNE), Bonn Germany; ^9^ German Centre for Neurodegenerative Diseases (DZNE), Bonn Germany; ^10^ German Center for Neurodegenerative Diseases (DZNE), Tübingen Germany; ^11^ Department of Psychiatry and Psychotherapy, Charité – Universitätsmedizin Berlin, corporate member of Freie Universität Berlin and Humboldt‐Universität zu Berlin‐Institute of Psychiatry and Psychotherapy, Berlin Germany; ^12^ Department of Psychiatry and Psychotherapy, Charité, Charitéplatz 1, Berlin Germany; ^13^ German Center for Neurodegenerative Diseases, Tübingen Germany; ^14^ German Center for Neurodegenerative Diseases (DZNE), Göttingen Germany; ^15^ Department of Psychosomatic Medicine, University Hospital Rostock, Rostock, Mecklenburg‐Vorpommern Germany; ^16^ Deutsches Zentrum für Neurodegenerative Erkrankungen e. V. (DZNE), site Rostock / Greifswald, Rostock, Mecklenburg‐Vorpommern Germany; ^17^ German Center for Neurodegenerative Diseases (DZNE), Rostock, Mecklenburg‐Vorpommem Germany

## Abstract

**Background:**

Inflammation is recognized as a key hallmark of Alzheimer’s disease (AD), alongside amyloid beta (Aβ) accumulation and tau pathology. Recent evidence suggests that neuroinflammatory markers in cerebrospinal fluid (CSF) are associated with progressive neurodegeneration and regional brain atrophy. In this study, we investigated the relationship between CSF inflammatory markers and atrophy in the basal forebrain and hippocampus longitudinally.

**Method:**

We included 296 participants (37 with AD dementia, 69 with mild cognitive impairment (MCI), 98 with subjective cognitive decline (SCD) and 92 healthy control) from the DELCODE study. Data included baseline CSF markers, the Aβ42‐phosphotau181 ratio, disease diagnosis, ApoE4 status, and longitudinal structural MRI volumes for specific brain regions, with a mean follow‐up of 19.8 months (SD = 16.7). Latent factors were previously derived via Bayesian confirmatory factor analysis from 14 CSF markers and classified into Synaptic, Microglia, Chemokine/Cytokine, and Complement groups. We used linear mixed‐effects models to assess interactions between latent factors and time on brain regions‐controlling for age, sex, education and ApoE4 status‐ and, based on our systematic review, examined whether neurogranin, sTREM2, ferritin, and YKL40 predicted longitudinal changes.

**Result:**

Our findings revealed significant interaction effects between specific biomarkers and regional brain atrophy. Longitudinal atrophy in the hippocampus was significantly associated with higher levels of the synaptic marker (β = ‐0.018, *p* = 0.004), sTREM2 (β = ‐0.012, *p* = 0.031), and YKL40 (β = ‐0.022, *p* = 0.0002). Similarly, increased levels of ferritin (β = ‐0.040, *p* = 0.024) and YKL40 (β = ‐0.041, *p* = 0.029) were predictive of longitudinal atrophy in the basal forebrain. In contrast, neurogranin and other latent factors—including microglia, chemokine/cytokine, and complement—did not show significant associations with atrophy in either brain region.

**Conclusion:**

These results suggest that certain biomarkers, particularly the synaptic latent factor and the individual markers sTREM2, ferritin, and YKL40, are predictive of longitudinal neurodegeneration in key brain regions vulnerable to Alzheimer's disease. The associations found with hippocampal and basal forebrain atrophy highlight the potential of these markers for tracking disease progression and improving early detection strategies.